# 

**Published:** 1888-11-17

**Authors:** 


CONTENTS.

**Mr. Gladstone as an Object Lesson** - - - 97

**In and Out Among the Hospitals** By a SECRETARY OF TEN YEARS' STANDING.—Royal Infirmary, Dundee, &c. - 98

**Sixteen Years of the Hospital Sunday Fund: Its Influence on the Metropolitan Medical Charities.** —By SIR SYDNEY WATERLOW, Bart. - - - - - 99

**The Editor's Letter-box.**—Village Homes, etc. - - 101

**The Doctor's Pulpit.** — The Seven Ages of Man—VII. "Sans Everything" - - - - - 102

**Voyages in Vacation.**—VIII. Cronstadt and St. Petersburg - 103

**Notes and News** - - - - - - - 104

**Everybody's Page** - - - - - - - 106

**Annotations.**—Processions or Puddings?—What is a Lunatic? —A Veritable New Cure - - - - - - 107

**How to Cultivate the Sense of Smell.**—By a CONSULTING PHYSICIAN - - - - - - - 108

**Physiology and its Uses.**—The Blood - - - - 109

**False Impressions.**—By CAROLINE FOTHERGILL - - 110

**Scraps and Gleanings** - - - - - - 112

**Amusements and Relaxation** - - - - - 112

EXTRA SUPPLEMENT.—THE NURSING MIRROR.

**En Passant**—Advance Australia.—Salop Infirmary.—In the Nurse's Absence.—News from Newark.—Mildmay Deaconesses.—Homœopathic Nurses.—Carelessness.—Wigan Infirmary.—King Stork - - - - - - - xxv
**Original Articles.**—Sick Nursing Among the Rich - xxvi
**Everybody's Opinion.**—Nurses and Presents—Holiday v. Convalescent Homes for Nurses - - - - - xxvii
**The Nurses' Bookshelf** - - - - - - xxviii
**Presentations—Appointments** - - - - - xxviii
**Vacancies—Notes and Queries** - - - - - xxxiii

